# Retraction Note: PIWIL2 restrains the progression of thyroid cancer via interaction with miR-146a-3p

**DOI:** 10.1186/s12902-025-01950-z

**Published:** 2025-05-12

**Authors:** Xiaoxiao Lu, Qingyun Zhu, Hong Du, Mingjun Gu, Xiangqi Li

**Affiliations:** 1 Department of Endocrinology and Metabolism, Punan Hospital, Pudong New Area, Shanghai, 200125 China; 2 Department of Intervention, Gongli Hospital, Naval Medical University, Shanghai, 200135 China; 3 Department of General Practice, Hudong Community Health Service Centre, Pudong New Area, Shanghai, 200129 China; 4 Department of Endocrinology and Metabolism, Gongli Hospital, Naval Medical University, Shanghai, 200135 China

**Retraction Note: BMC Endocrine Disorders (2023) 23:184**

10.1186/s12902-023-01416-0

The Editor has retracted this article because the authors were not able to provide appropriate documentation of approval from the relevant ethics committees.

Authors disagree with the retraction of the paper.

